# Smart Health System to Detect Dementia Disorders Using Virtual Reality

**DOI:** 10.3390/healthcare9070810

**Published:** 2021-06-28

**Authors:** Areej Y. Bayahya, Wadee Alhalabi, Sultan H. AlAmri

**Affiliations:** 1 Department of Computer Science, King Abdulaziz University, Jeddah 21589, Saudi Arabia; wsalhalabi@kau.edu.sa; 2 Virtual Reality Research Group, King Abdulaziz University, Jeddah 21589, Saudi Arabia; 3 Department of Family Medicine, King Abdulaziz University, Jeddah 21589, Saudi Arabia; shalamri1@kau.edu.sa

**Keywords:** virtual reality, smart health, dementia, geriatric medicine, visuospatial, memory

## Abstract

Smart health technology includes physical sensors, intelligent sensors, and output advice to help monitor patients’ health and adjust their behavior. Virtual reality (VR) plays an increasingly larger role to improve health outcomes, being used in a variety of medical specialties including robotic surgery, diagnosis of some difficult diseases, and virtual reality pain distraction for severe burn patients. Smart VR health technology acts as a decision support system in the diseases diagnostic test of patients as they perform real world tasks in virtual reality (e.g., navigation). In this study, a non-invasive, cognitive computerized test based on 3D virtual environments for detecting the main symptoms of dementia (memory loss, visuospatial defects, and spatial navigation) is proposed. In a recent study, the system was tested on 115 real patients of which thirty had a dementia, sixty-five were cognitively healthy, and twenty had a mild cognitive impairment (MCI). The performance of the VR system was compared with Mini-Cog test, where the latter is used to measure cognitive impaired patients in the traditional diagnosis system at the clinic. It was observed that visuospatial and memory recall scores in both clinical diagnosis and VR system of dementia patients were less than those of MCI patients, and the scores of MCI patients were less than those of the control group. Furthermore, there is a perfect agreement between the standard methods in functional evaluation and navigational ability in our system where P-value in weighted Kappa statistic= 100% and between Mini-Cog-clinical diagnosis vs. VR scores where P-value in weighted Kappa statistic= 93%.

## 1. Introduction

Computers play a vital role as a decision support system in the diseases’ diagnostic test. One of the most promising computerized methods is to measure patients cognitive performance as they perform real world tasks in virtual reality (e.g., navigation) [1,2]. Virtual reality (VR) is already making a difference in the health care industry. It has been harnessed to provide a more appropriate and effective health care service in several health care services including assisting doctors, surgeons, physicians, and nurses in-surgery; hands on surgical simulators for training; diagnosis of diseases; physical treatment; and long-term condition management. Where diagnosing disease is concerned, system smart health can offer a method of monitoring health levels or improving health outcomes at the clinic.

According to the National Institutes of Health [1], dementia is not a specific disease, it is a descriptive term of defects in the human brain, which leads to a collection of symptoms of neurocognitive dysfunction [1]. In addition to age, the strongest risk factors for dementia include chronic health conditions such as hypertension and diabetes, unhealthy lifestyle such as smoking, and family medical history [1]. Dementia can progress rapidly and, in a span of less than two years, can advance from the first symptoms to the next stage. The symptoms of disease appear as forgetting appointments, getting lost, impairment in receiving and recalling new information, having impaired visuospatial skills, poor judgment such as trouble managing finances, having impaired language function, behavior changes such as social withdrawal, and failure in usual activities such as making coffee and driving a car [1].

**Figure 1:**
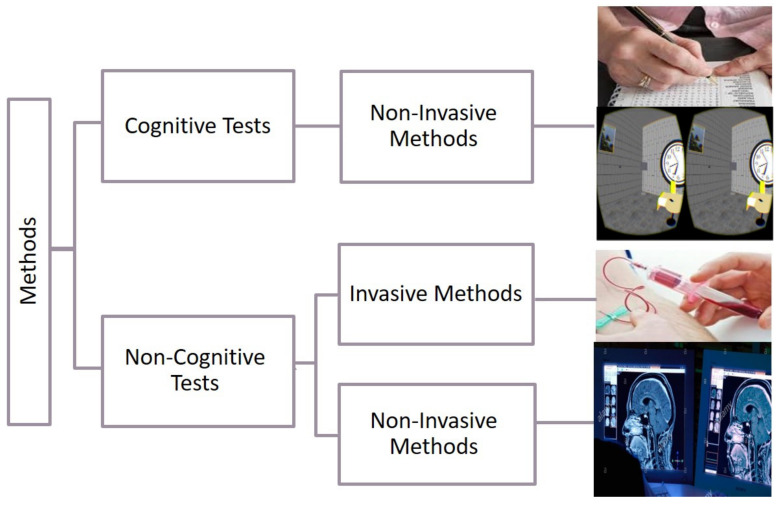
Classification methods to detect cognitive impairment [2].

The detection methods of cognitive impairment are classified into two classes: cognitive and non-cognitive tests (see Figure 1) [2]. Cognitive tests are procedures based on measuring the patient’s cognition through the use of questions, tasks, and problem-solving activities. These procedures are considered non-invasive methods. They are easy to implement, accurate, and harmless [2]. Invasive methods depend on information and data taken from inside the human body through procedures such as blood extraction, surgery, and drug treatments (see Figure 1). Non-invasive methods are medical procedures not requiring the introduction of instruments into the body (e.g., MRI scans). It is suitable for the majority of patients [2]. For example, AD requires several stages to be diagnosed, and the stage of diagnosis can include both cognitive and non-cognitive tests. Accordingly, most studies include non-cognitive methods [3,4,5,6] concurrently with a cognitive test [7,8,9]. 

One of the non-cognitive methods to diagnose Alzheimer’s disease is by incorporating biomarker tests. Numerous noncognitive tests are available [3,4,5,6], but no test so far has shown the preference in all criteria and inevitable result of medical research to diagnose Alzheimer’s disease. The cause is that the biological outcomes of Alzheimer’s patients are very similar to other diseases. 

For the detection of dementia, several cognitive functions need to be assessed. These include visuospatial disorientation, complex attention, executive function, learning and memory, language, perceptual-motor, or social cognition [1]. Bayahya et al. [10] focused on visuospatial disorientation and memory dysfunction to indicate function deficits. Cognitive tests based on visuospatial dysfunction for elderly reveal the symptoms of Alzheimer’s disease for up to five years before the onset of other symptoms, including memory impairment [11].

The most common method used to detect dementia and Alzheimer’s disease is cognitive testing. These tests have been relied upon for a long time in their standard form (pen–paper test). Then, there was a gradual introduction of technical and software approaches in the medical field, especially for mental illnesses such as AD [12]. One of the most recent developments in the technology is the use of VR-based assessment functionality to detect Alzheimer’s disease. The Standard Cognitive Test is known as the standard neuropsychological assessment. It depends on questions and tasks that require solving various problem to diagnose patients of Alzheimer’s disease. Sensitive cognitive tests should be selected to detect cognitive impairment in mild stages of AD. Numerous cognitive tests are available [7] that observe the patient’s performance in problem-solving, planning, spatial navigation, memory and recall items, and defining and formulating goals. The Mini-Cog test [13] is among the most reliable for people who are not influenced by education or language. The Mini-Cog test is the traditional diagnosis system used to measure cognitive impairment in the clinic. It tests two different aspects of cognitive domains: visuospatial disorientation and memory recall. The Mini-Cog test is used for enhancing and validating primary care and is available in multiple languages/cultures [14]. It is useful when used in contexts where there are little or no biases in education, language, or race. Furthermore, it has a short administration time. Arabic versions of Mini-Cog test [15] have shown that the test is valid and concise; it gives satisfactory screening results of cognitive deficits. The test can be used to increase detection of cognitive impairment in older adults. The Mini-Cog gives 99% sensitivity, 93% specificity, and 96% diagnostic value. Standard cognitive or neuropsychological tests lack an important aspect, which is measuring cognitive impairment in the real world; also, they have low ecological validity. This leads to excluding the contribution of important sensorimotor aspects. In addition, these tests do not provide an adequate evaluation of memory for routes and spatial navigation. They lack large-scale navigation to detect navigation impairment and have limited generalizability to real-world function to measure memory routes [16,17]. It is worth mentioning that some of the patients in the early stages of Alzheimer’s perform well on standard cognitive tests, but in fact, they have functional disabilities in their daily lives. Furthermore, these tests cannot be performed on illiterate and uneducated people [9]. 

One promising method in the medical field, especially for diagnosing dementia and Alzheimer’s diseases, is using VR technology. Different innovative ways of using VR for diagnosing early stages of Alzheimer’s disease in order to avoid the weaknesses of traditional tests were suggested by [16,17,18,19,20,21,22]. Their proposals are focused on two important domains: navigational processes or memory processes or both. They try to concentrate on assessing cognitive impairment in spatial navigation as well as memory deficit cases. A real-world navigation test is compared with a virtual reality version in the study done by Cushman et al. [22]. Spatial orientation is investigated by Tu et al. [21] by using a novel ecological, non-immersive virtual supermarket task. In order to examine age- and Alzheimer’s disease-related differences in route learning and spatial memory, an immersive virtual city was created by Zakzanis et al. [16]. 

In a link with above mentioned studies, Lesk et al. [18] and Plancher et al. [20] assume that memory deficits may inhibit navigation within a virtual environment. Subsequently, Lesk et al. [18] and Plancher et al. [20], who focus on memory assessment to diagnose the disease, suggested a significant correlation between daily memory complaints and performance of VR test. To measure topographical memory (TM) in a non-immersive virtual town, three novel tests were created by Pengas et al. [19].

Additionally, the researchers chose to use a non-immersive virtual reality rather than using full immersion, because it was observed that the latter possess some problems for patients. It is important to notice that all previous studies have used statistical criteria in analyzing their results. Further to that, traditional cognitive tests were applied to compare the above works in order to emphasize the results for the AD diagnosis. 

The objective of a study by Bayahya et al. was to build a cognitive tool using VR environment in order to detect memory loss, visuospatial defects, and spatial navigation in patients with dementia. To this aim, four tasks were designed to assess two different cognitive domains: visuospatial task (spatial navigational, spatial orientation, and visual memory tasks) and memory tasks (delayed recall). After collecting scores from tasks, data were analyzed using nonparametric statistical tests including Wilcoxon signed-rank test [23], Cohen’s kappa [24], and Kruskal–Wallis H-Test [25]. The participants belonged to three different groups of elderly people: dementia, MCI, and health older. Dementia and MCI can have both deficits in one or more cognitive domains; however, MCI patients are independent related to the activities of daily living. In the work of Bayahya et al. [10], several objectives were considered:

To develop a VR environment along with statistical methods in order to help physicians detecting abnormality in behavior and perception caused by dementia.

- To use the statistical methods along with VR technology in order to evaluate the possibility of detecting visuospatial and memory deficits in patients who have dementia.
- To use simulated environment that tests two cognitive domains, memory and visuospatial deficits, for diagnosing patients with cognitive impairment.
- To compare the performance of participants who have early and moderately severe dementia, mild cognitive impairment (MCI), and older adults with normal cognitive functioning.
- To collect data from real patients while they performed common everyday tasks in virtual reality.

## 2. Methods and Materials

**Figure 2:**
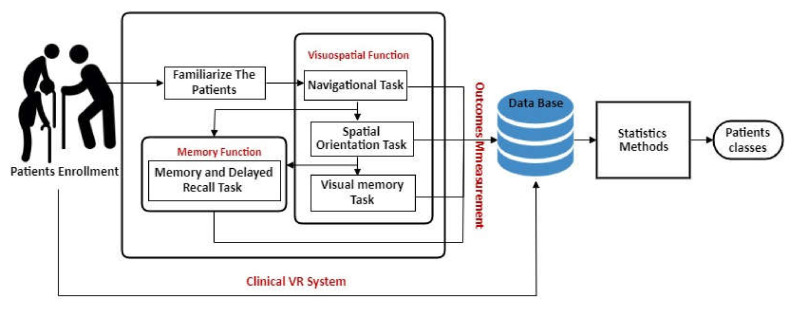
Architecture model of diagnosis dementia patients.

This study proposes a 3D serious game containing a model for patient information storage and retrieval, and cognitive test-based VR System with semi-immersive type of methodology. The scope was to determine whether the patient has cognitive impairment using a set of VR measures and statistical analysis. In addition, a comparison between VR system participants’ performance (that have MCI, early, and moderately severe dementia, and older adults who have a normal cognitive condition) and their performance on Mini-Cog test was performed. This model has been designed for cognitive impairment patients, both the educated or non-educated class, female or male. Additionally, the platform can be used in any neurology or clinical facility as a quantitative assessment of patients along with other cognitive or non-cognitive approaches. In the subsections that follow, we will discuss the architecture model used to classify dementia patients as shown in Figure 2.

### 2.1. Clinical and Demographic Information

**Figure 3:**
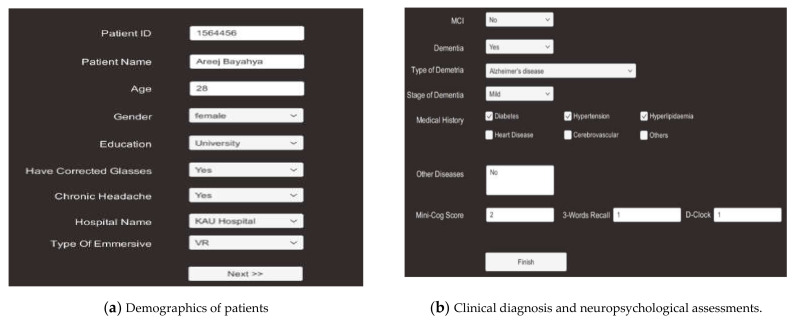
Demographics of clinical and medical history, clinical diagnosis, and neuropsychological assessments.

Creating new patient record is the first step before starting the VR system to generate a record that can be used for all subsequent patients’ outcome measures. In the proposed model, the physicians have to register the patient by providing personal information, vision impairment, patient’s history, medical history, and clinical diagnosis to the system, as shown in Figure 3.

### 2.2. The Virtual Scenario

To start, the VR system displays an automatic tour around the environment to prepare the patient for the test, where the patient captures the visible scenes in his memory. As the same time, he/she listens to the instructions of the system. The VR environment consists of a street overlooking the sea, and there are some shops and a supermarket. Then, the patient undergoes two cognitive tests: visuospatial function and memory function; each test produces cognitive scores. The data are compiled and the system derives evaluation performance. The system uses these data to detect the cognitive impairment of the patient. Each test contains tasks, specific scenarios through which cognitive testing is performed:

- Creating the patient record on the system.
- Starting the first task of testing.
- Calculating the scores.
- Repeating step 2 and step 3 for all tasks.
- Collecting all tasks scores measured in step 3 for all tests.
- Extracting outcome measures.
- Checking the results.

#### 2.2.1. Visuospatial Function

The visuo-spatial function is conceptualized in different domain aspects: visual perception, construction, and visual memory [26].

#### 2.2.2. Visual Perception, Spatial Orientation, and Topographical Orientation

**Figure 4:**
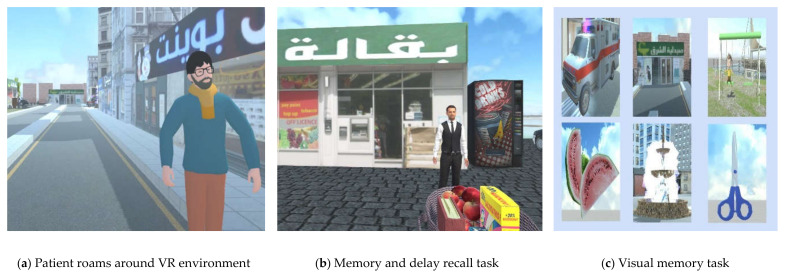
VR system to diagnosing dementia patients.

Navigational task and spatial orientation are a measure of the topographical orientation, judgment of direction and distance [15]; the patients need to correctly identify the path using input device (a joystick) that employs four directions (right, left, front, back) to move the avatar, on a big screen to immerse the patient in the VR environment. (Figure 4a). The tasks are performed in the following steps:

- Show video to the patient from start to destination.
- Patient sees the path from start point to destination.
- Start tasks with instructions.
- System checks if participant can navigate in the system.
- System calculates the patient’s path coordinates.
- System displays several questions to measure judgment of direction, which the patient verbally answers; then, the assistant/nurse uses the keyboard to enter the response directly at the same time. The system will check if the participant reaches the final destination.

#### 2.2.3. Visual Memory

The visual memory tasks are measures of visual information or (recognition) recall and topographical memory (Figure 4c). In this case, topographical memory includes encoding and perception of spatial orientation needed to walk in the surrounding environment [11]. This task is a part of the VR navigation where the items were displayed in a dedicated screen. Topographical recognition memory tests based on stimuli are pictures of scenes containing distinctive objects and in some items, people [27]. The task is performed in the following steps:

- Displays several images.
- Check if the patient remembers any of these images.

### 2.3. Memory Function

Another domain is memory function; it is the predominant cognitive domain used to detect dysfunctions associated with dementia disease. Memory and delay recall tasks measure memory deficits in patients with dementia by using a three-word recall algorithm [17] (Figure 4b). The task is performed in the following steps:

- System asks him/her to repeat three words.
- Check if patient navigates to store.
- Check if patient remembers the previous three words.

### 2.4. Outcomes Measurements

**Figure 5:**
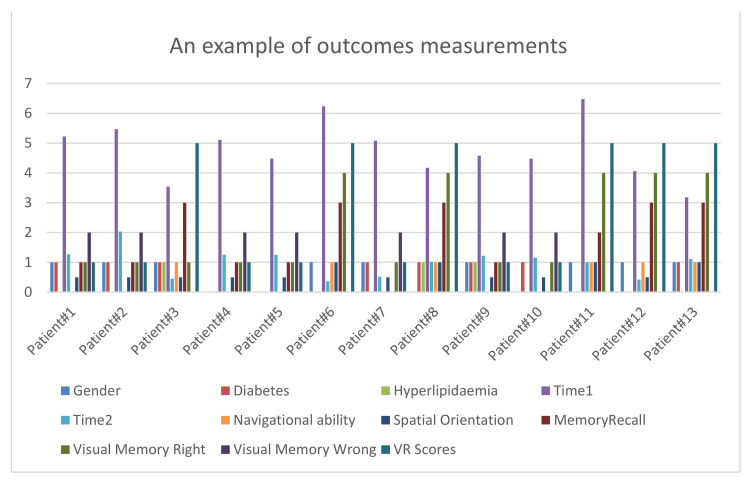
An example of outcomes measurements.

There are several factors that are assessed to detect cognitive impairment:

- Number of times the patient changed direction, the total time it took to arrive to the destination (Time1), and total time it took to finish the visual memory task (Time2) were recorded.
- Patient’s history and medical history including past head injuries or exposure to solvents, diabetes, hypertension, hyperlipidemia, checking if patient has dementia or MCI, type of dementia, stage of dementia, and functional evaluation. Patient’s history will be provided to the system before patient starts the VR test (see Figure 3).
- VR scores includes navigational ability, spatial orientation, memory recall, visual memory correct, and incorrect visual memory (see Figure 5).

## 3. Result

The experimental was applied on 115 real patients from Dr. Soliman Fakeeh Hospital, King Abdul-Aziz Hospital, International Medical Centre, and Association of Elderly People Friends. Thirty had dementia, sixty-five were cognitively healthy, and twenty had MCI. All patients were normal elderly people aged 50 or older and came from both the educated and non-educated class. All participants signed a consent form before the test began. The medical team, represented by the nurse and assistant, did a simple explanation for the participants, and trained them to hold the joystick. All groups of participants completed the test without fear or any withdrawal. As observed from the experiment, dementia patients took slightly more time adapting to the environment than the others. Moreover, they spent slightly more time completing the test than the others. Our VR test is user friendly, easy to use, inexpensive, and less time-consuming, as all groups took less than 5 min.

### 3.1. Pre-Processing Data

**Figure 6:**
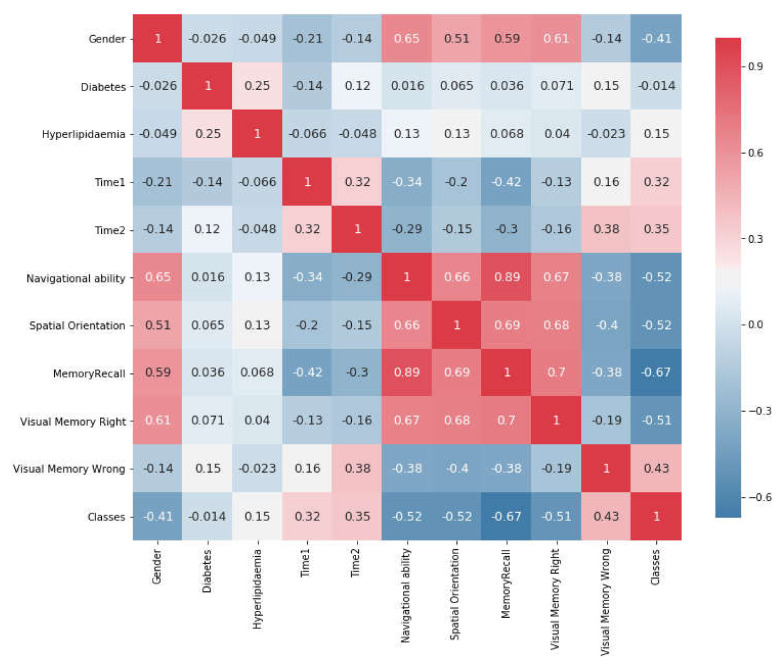
Correlation Matrix Plot Between Attributes Features.

The proposed system used different software and programming languages: Unity (KAU, Jeddah and Saudi Arabia), Jupyter Notebook (KAU, Jeddah and Saudi Arabia), as programs dealing with C#, Python (KAU, Jeddah and Saudi Arabia), and Java Script (KAU, Jeddah and Saudi Arabia). First, the (.CSV) file is read and Pandas library is imported to deal with the csv data (See Figure 5). Secondly, the non-numerical data elements are converted into numerical formats. Then, feature selection is applied to extract the most important attributes depending on Heatmap (Figure 6). Heatmap gives values that describe the correlation between two different features.

Before using statistical methods, we need to preprocess the data and find the relationship between the features. Therefore, this study applied feature selection by using Heatmap. Figure 6 shows the changes between two features on a Correlation Matrix Plot as a heatmap with values between −1 and 1. When the value is close to 1, it demonstrates a positive strong correlation between these attributes and a larger correlation magnitude, whereas, when the value is close to −1, it shows a negative strong correlation between the same attributes. For example, VR system (Bayahya [10], Jeddah and Saudi Arabia), memory recall vs. navigational ability appear to have a larger correlation magnitude (0.9) to each other.

Similarly, there is a strong correlation between spatial orientation vs. visual memory correct (0.68) as well as spatial orientation vs. VR system memory recall (0.7). In contrast, there is a negative strong correlation between visual memory right vs. time consumed in first task (−0.13)

As the nature of data is having non-parametric and non-normal distribution, non-parametric statistical methods were used to assess the agreement between clinical scores and VR system scores to determine if there are any statistically significant differences between participants. As the data is labelled and for the purpose of applying statistical methods, all data will be split into three classes: those who have cognitive impairment (dementia), those who are cognitively healthy, and those who have MCI.

For comparing the agreement between clinical diagnosis results and VR system, Equation (1) was used to compute the VR scores of the patients and make them compatible with the result of the Mini-Cog test.

Score = (Navigational ability + Spatial orientation + Memory recall + Visual memory correct − Visual memory incorrect)/2(1)

### 3.2. Statistical Methods

Since analyzing the performance results through different techniques indicates high reliability, we have used statistical methods including Wilcoxon signed-rank test [23], Friedman Test [28], Cohen’s kappa [24], and Kruskal-Wallis H-Test [25]. 

Wilcoxon signed-rank test [23] and Cohen’s kappa [24] were used to assess the agreement between clinical scores and the VR system scores. The objective was to test if there is a significant difference in dependent variable scores, and to determine whether the distribution of the difference scores is symmetric about zero. The Friedman test is another non-parametric statistical alternative to the one-way ANOVA that compares two or more dependent paired samples in one group [28]. It calculates and analyzes the difference between classification on standard Mini-Cog Scores vs. classification on VR Scores vs. classification on clinical diagnosis. Kruskal-Wallis H-Test was used to determine if there are statistically significant differences between participants who have early and moderate severe dementia, MCI, or normal cognitive ability.

VR system and neuropsychological clinical test results were analyzed using Wilcoxon signed-rank test and Cohen’s kappa. The latter test measured inter-rater reliability between-subject factor, i.e., interobserver agreement, whereas Wilcoxon signed-rank test calculated the difference between each set of pairs and analyzed these differences. Cohen’s kappa assessed the agreement between alternative methods of categorical variables, which are clinical scores vs. VR system scores and function evaluation clinic vs. function evaluation VR system. Cohen’s kappa is especially used when new techniques are under study. As shown in Table 1, Kappa values have a range between (0 ≤ κ ≤ 1) and a maximum of 1 when agreement is perfect. Weighted Kappa statistic is used with ordinal categories and measures the degree of agreement. 

Wilcoxon signed-rank is a nonparametric statistical test that compares two dependent matched paired groups and calculates and analyzes the difference between clinical diagnosis scores vs. VR system scores and function evaluation clinical diagnosis vs. VR system navigational ability. It can be used as an alternative to paired t-test for dependent samples, and it tests the null hypothesis (two related paired samples come from the same distribution).

Differences in participant groups’ demographics and performance on cognitive tests were analyzed using the Kruskal–Wallis H-test. It was analyzed between groups to assess the differences among the group (control, MCI, dementia patients). It is a non-parametric version of one-way ANOVA on ranks, used for two or more independent sample groups on a single, non-normally distributed variable and ordinal scale dependent variables [25]. Kruskal–Wallis H-test assumption for null hypothesis is that the population median of all the groups are equal. 

## 4. Discussion of Results

### 4.1. Demographics of Clinical and Neuropsychological Assessments

The levels of patient history, age, education, and gender were included as control variables in all statistical analyses. Demographics and baseline scores for all groups are shown in Table 2. Differences in participant group demographics and performance on cognitive tests were assessed using Kruskal–Wallis H-Test, Cohen’s kappa, and weighted kappa coefficient, as shown in Table 2.

All participants are above 60 years in age (60 ≤ μ ≤ 80); the dementia patients belong to the highest age group. Hyperlipemia is significantly different in participant groups where P-value equals to 0.00090. The mean rank for hyperlipemia of dementia patients (μ = 0.06) was less than that of MCI patients and of the control group. In contrast, there are no significant differences in participant groups regarding diabetes, where P-value was equal to 0.4.

In addition, there are differences in participant group performances on Time-Task 1 and Time-Task 2, where *p*-value < 0.05. As it can be observed from Table 2, there are no significant differences between dementia patients and MCI in accomplishment time in Task 1 and Task 2.

### 4.2. Memory Recall as Diagnostic Predictors of Dementia and MCI 

Memory recall performance on the experimental task and clinical diagnosis was measured using Kruskal–Wallis H-test in within-subject groups. There are significant differences in the participant groups for all alternative methods. In clinical diagnosis, memory recall function scores are significantly different in participant groups where *p*-value < 0.001. As it can be observed form Table 2, the same performance results are noticed in VR system where there are significant differences in participant groups *p*-value < 0.001. Overall, the P-value is below the significant level alpha = 0.05, leading to the conclusion that the null hypothesis is rejected, and the samples drawn from populations have different distributions. 

**Figure 7:**
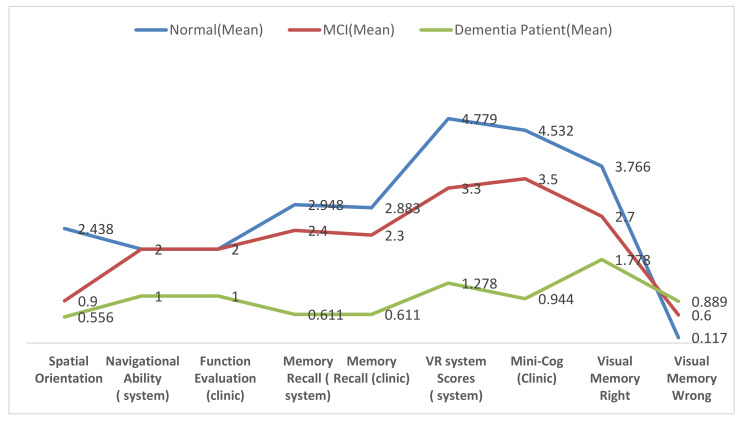
Means of cognitive test and neuropsychological abilities.

As shown in Table 2 and Figure 7, the mean rank for memory recall scores in both clinical diagnosis and VR system of dementia patients was less than that of MCI patients, and that of MCI patients was less than that of the control group. This concludes that the distributions of all the groups are not equal. 

**Figure 8:**
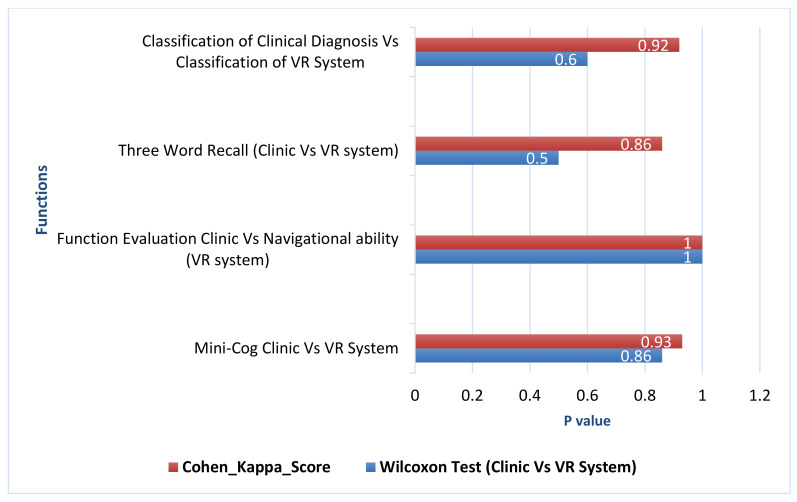
Wilcoxon test (*p*-Value) and Cohen kappa of cognitive test in both clinic vs. vr system.

Memory recall performance in the experimental task and clinical diagnosis were compared using Wilcoxon signed-rank test between dependent-subject groups. It compared all participants in two alternative methods to test if these two samples come from the same distributions. As shown in Table 3, there are no significant differences between each set of pairs where *p*-value = 0.500 and both clinical diagnosis and VR system have a mean equal to 2.4 (SD = 0.9). The agreement between alternative methods in memory recall is measured using weighted kappa statistics. As observed in Table 3, there is a perfect agreement between the alternative methods in all participants where *p*-value in Kappa statistic = 0.86 (see Figure 8). This concludes that this experiment failed to reject the null hypothesis that is paired samples have the same distribution (fail to reject H0). Therefore, sample distributions are equal, and the differences between them is symmetric about zero.

### 4.3. Functional Evaluation and Navigational Ability

As was statistically observed, there is a strong relationship between functional evaluation performance on clinical diagnosis and the navigational ability on the experimental task. On the navigational ability task, all dementia patients could not reach the final destination versus the other groups that reached to the final position. This concluded that all dementia patients had functional impairment.

Functional evaluation performance and navigational ability were assessed using Kruskal–Wallis H-test between groups. Concerning their functional evaluation scores, the dementia patients were impaired in comparison with the control population and MCI. As observed in Table 2, there are differences in performance on functional evaluation and navigational ability in dementia class and other classes where *p*-value < 0.001. All control and MCI patients have intact function where the mean rank for them was μ = 2 (SD = 0); however, all dementia patients have impaired function where the mean was μ = 1 (SD = 0), indicating that the populations have different distributions.

The navigational ability performance in experimental task and the functional evaluation in clinical diagnosis were compared using Wilcoxon signed-rank test within-subjects. Both clinical diagnosis and VR system have mean rank μ = 1.8 (SD = 0.37). As observed in Table 3, there were no significant differences between each set of pairs where *p*-value = 1. This indicates that the null hypothesis that is paired samples have the same distribution (fail to reject H0) is confirmed. Sample distributions are equal and the differences between them is symmetric to approximately zero. 

To assess the agreement of functional evaluation between VR system and clinical diagnosis, Cohen’s kappa statistics were used. As shown in Table 3 and Figure 7, *p*-value in Kappa statistic = 1, which means a perfect agreement between the alternative methods of measured functional evaluation.

### 4.4. Spatial Orientation Performance as Diagnostic Predictors of Dementia and MCI

Spatial orientation performance on the experimental task was assessed using a Kruskal–Wallis H-test. It used within-subject groups to compare spatial orientation performance between three different groups. In the two groups of interest, MCI showed a better cognitive profile (μ = 0.900) than dementia (μ = 0.500) on spatial orientation (see Table 2). Overall, spatial orientation scores in the VR system for dementia patients were less than those of MCI patients, and those of MCI patients were less than those of the control group (see Figure 7). As it can be observed from Table 2, the population of all the groups were not equal (*p*-value < 0.001). This rejects the null hypothesis that the difference is due to random sampling and indicates that the populations have different distributions because *p*-value < 0.05 is too small.

### 4.5. Visual Memory

Visual memory function has two tests: visual memory correct and visual memory incorrect. Visual memory correct and visual memory incorrect scores were assessed using Kruskal–Wallis H-test. There are significant differences in performance between all groups in both tests. In visual memory right, there is a difference in performance among the groups where *p*-value < 0.001. As shown in Table 2, the mean rank of correctness choices for dementia patients (μ = 1.7) was less than that of MCI patients (μ = 2.7), and that of MCI patients was lower than that of the control group (μ = 3.7). This indicates that the population means of all the groups are not equal, and the populations have different distributions (see Figure 7).

On the other hand, as shown in Table 2, the visual memory incorrect test has *p*-value < 0.001 among the groups. As it can be observed from Figure 7, the mean rank of faultiness choices for dementia patients (μ = 0.889) was higher than that of MCI patients (μ = 0.600) and that of MCI patients was higher than that of the control group (μ = 0.11), thus rejecting the null hypothesis, because the samples were drawn from populations with differing distributions.

### 4.6. Visuospatial Function Using Clinical Scores and VR Scores as a Diagnostic Predictor of Dementia and MCI 

Visuospatial function was tested by using Mini-Cog test and VR scores. They were assessed in both the experimental task and clinical diagnosis by using Kruskal–Wallis H-test. As shown in Table 2, the mean rank for Mini-Cog scores in clinical diagnosis and VR scores of dementia patients was lower than that of MCI patients, and that of MCI patients was lower than that of the control group. In clinical diagnosis, there are significant differences between groups—dementia patients have μ = 0.944, MCI patients have μ = 3.500, and control group patients have μ = 4.532. As shown in Figure 7, the VR system has significant differences between groups—dementia patients have μ = 1.28, MCI patients have μ = 3.3, and control group patients have μ = 4.78. In both alternative methods, there are significant differences in the participant groups. The *p*-value in VR system < 0.001, whereas the same performance result in clinical diagnosis has *p*-value < 0.001. Consequently, *p*-value in both alternative methods is below the significance level alpha = 0.05, thereby rejecting the null hypothesis and indicating that the samples are drawn from populations with differing distributions. This concluded that the distributions of all groups are not equal.

Mini-Cog test performance in clinical diagnosis and VR scores in experimental task were assessed using Wilcoxon signed-rank test (within subject). It compared all participants in two alternative methods to test if these two samples come from the same distributions. As shown in Table 3, there were no significant differences between each set of pairs where *p*-value = 0.860. In addition, both clinical diagnosis and VR system have the equal ranked mean of the same samples μ = 3.8. Furthermore, as can be observed from Figure 8, there is a perfect agreement between the alternative methods in all participants where *p*-value in weighted Kappa statistic= 0.93. Similarly, the classification performance in clinical diagnosis and VR system were assessed using Wilcoxon signed-rank test. As it can be observed from Table 3, there were no significant differences between each set of pairs where *p*-value = 0.6, and there is a perfect agreement between the alternative methods where *p*-value in Kappa statistic= 0.92. This indicates that this experiment failed to reject the null hypothesis that paired samples have the same distribution (fail to reject H0); sample distributions are equal, and the differences between them are symmetric to approximately zero. 

This study was used to measure the patients’ classification using three different methods: patients’ classification using standard Mini-Cog test, patients’ classification using standard Mini-Cog test with evaluation function, and patients’ classification using VR system with navigational ability. Patients’ classification performance was assessed using Friedman test between dependent-subject groups. As observed in Table 4, there is a perfect agreement between all the alternative methods in all participants where *p*-value in Kappa statistic= 0.90. Furthermore, there were no significant differences between each set of all pairs of samples where *p*-value = 0.180. This indicates that the experiment failed to reject the null hypothesis; that is, all paired samples have the same distribution; therefore, all sample distributions are equal, and the differences between them are symmetric to approximately zero.

As observed, Mini-Cog scores vs. VR system scores, and functional evaluation vs. navigational ability in both clinical diagnosis and VR system have the same distribution and have very good agreement between scores. Additionally, cognitive tests scores in both clinical diagnosis and VR system of dementia patients were less than those of MCI patients, and those of MCI patients were less than those of the control group. 

This study compares the classification accuracy of all participants using traditional clinical diagnosis method vs. the VR and statistical methods. Dementia diagnosis at clinic (expert diagnosis) depends on functional evaluation plus a cognitive test such as Mini-Cog test at early stages of disease. In this experiment, it was observed that patients’ classification at clinic, which depended on the Mini-Cog test with functional evaluation, showed 94% accuracy, whereas VR system combined with navigational ability showed 97.22%. 

## 5. Conclusions 

As demonstrated above, with a smart dementia platform, combining a model for patient information storage and retrieval and cognitive test-based VR system, it is possible to classify and detect cognitive impairment in patients with dementia. This becomes a powerful tool that can be used in early detection of dementia, so that timely intervention measures can be applied. This study addresses these issues as well as solves several problems that are associated with currently prevailing clinical practices, as mentioned below:

- The limited diagnosis of spatial orientation problems in the clinic—the limitation is due to lack of feasible and proper practical tasks in a clinical setting;
- The assessment must reflect real-world conditions for accurate assessment of functional disability in dementia;
- Diagnosis of dementia consumes time, effort, and high cost, especially in the early stages of the disease.

Since there is a need to use advanced tools to assess and detect functional cognitive impairment, the proposed system can be considered an excellent substitute for prevailing/current diagnostic methods related to cognitive tests. 

This study focused on a serious game based on semi immersive cognitive tools to detect memory loss, visuospatial defects, and spatial navigation ability in patients with dementia. VR system contains different tasks to measure deficit of patients. As classically observed, Mini-Cog scores vs. VR system scores, functional evaluation vs. navigational ability, and memory recall scores in both clinical diagnosis and VR system had the same distribution and had very good agreement between scores. Moreover, visuospatial tests scores and memory recall scores in both clinical diagnosis and VR system of dementia patients were less than those of MCI patients, and those of MCI patients were less than those of the control group. Furthermore, spatial orientation function and visual memory right in VR system of dementia patients were less than those of MCI patients, and those of MCI patients were less than those of the control group. This concluded that the populations of all the groups were not equal, confirming the previous result where memory recall function scores, visual memory, spatial orientation, Mini-Cog Test, and VR Scores were significantly different in within-subject groups with *p*-value <.05. In another aspect, all dementia patients had functional impairment vs. the other groups that had intact functioning in both clinical diagnosis and VR system. 

## Figures and Tables

**Table 1:** Brief summary of common standard cognitive tests for dementia and AD diagnosis.

Kappa Statistic	Agreement
<0.20	Poor
<0.40	Fair
<0.60	Moderate
<0.80	Good
to 1	Very good

**Table 2:** Kruskal–Wallis H-Test, means, and SDs of demographics and general neuropsychological abilities and VR system.

Function/Group	Normal (Mean, Stdv)	MCI (Mean, Stdv)	Dementia Patient (Mean, Stdv)	Kruskal–Wallis H-Test (Normal vs. MCI vs. Dementia)	Hypothesis Result
Age (years)	(60, 7.85)	(68.5, 9.34)	(80, 8.08)	---	---
Sex (M/F)	(10:55)	(8:12)	(20:10)	---	---
Level of education	(1, 1)	(1, 1)	(1.5, 1.5)	---	not significant
Diabetes	(0.58, 0.49)	(0.74, 0.44)	(0.56, 0.49)	0.44	Same Distribution
Hyperlipemia	(0.17, 0.37)	(0.52, 0.49)	(0.06, 0.24)	<0.001	Different Distributions (Reject H0) The population median of all the groups are not equal.
Time-Task 1 (min)	(3.70, 0.79)	(4.02, 0.46)	(4.57, 0.89)		
Time-Task 2 (min)	(0.62, 0.43)	(1.26, 0.49)	(1.06, 0.52)		
Navigational Ability-VR	(2, 0)	(2, 0)	(1, 0)		
Function Evaluation-Clinical	(2,0)	(2,0)	(1,0)		
Spatial Orientation-VR	(2.44, 0.98)	(0.90, 0.20)	(0.55, 0.49)		
Memory Recall-VR	(2.95, 0.22)	(2.40, 0.49)	(0.61, 0.68)		
Memory Recall-Clinical	(2.88, 0.32)	(2.30, 0.64)	(0.61, 0.89)		
VR system Scores	(4.78, 0.53)	(3.3, 0.64)	(1.28, 1.36)		
Mini-Cog Test-Clinical	(4.53, 0.62)	(3.50, 0.67)	(0.94, 1.22)		
Visual Memory Right	(3.77, 0.60)	(2.70, 0.64)	(1.78, 1.58)		
Visual Memory Wrong	(0.12, 0.42)	(0.60, 0.80)	(0.89, 0.88)		

**Table 3:** Wilcoxon test and Cohen kappa of cognitive test in both clinic vs. VR system.

Function/Group	All Participants (m/stdv)	Wilcoxon Test (Clinic vs. VR System)	Hypothesis Result	Cohen_Kappa_Score
Mini-Cog Clinical	(3.81, 1.54)	*p* = 0.860	Same distribution (fail to reject H0)	0.93
VR System (Scores)	(3.83, 1.58)			
Function Evaluation-Clinical	(1.82, 0.377)	*p* = 1.000		1
Navigational ability-VR	(1.82, 0.377)			
Three Word Recall-Clinical	(2.43, 0.98)	*p* = 0.500		0.86
Three Word Recall-VR	(2.49, 0.95)			
Classification of Clinical	(0.32, 0.62)	*p* = 0.6		0.92
Classification of VR System	(0.25, 0.53)			

**Table 4:** Friedman test and Cohen kappa of cognitive test in clinical diagnosis vs. VR system vs. standard Mini-Cog test.

Function/Group	All Participants (μ/std)	Friedman Test (Clinical vs. VR vs. Standard Mini-Cog Test)	Hypothesis Result	Cohen Kappa Score
Classification of Clinical diagnosis	(0.32, 0.62)	*p* = 0.1800923	Same distribution (fail to reject H0)	0.90
Classification of Standard Mini-Cog Scores	(0.28, 0.58)			
Classification of VR Scores	(0.25, 0.53)			

## Data Availability

The data presented in this study are available on request from the corresponding author. The data are not publicly available due to privacy.
